# A pulmonary rehabilitation program is an effective strategy to improve forced vital capacity, muscle strength, and functional exercise capacity similarly in adults and older people with post-severe COVID-19 who required mechanical ventilation

**DOI:** 10.1186/s12877-024-04910-9

**Published:** 2024-04-04

**Authors:** Rodrigo Muñoz-Cofré, María Fernanda del Valle, Gabriel Nasri Marzuca-Nassr, Jorge Valenzuela, Mariano del Sol, Constanza Díaz Canales, Pablo A. Lizana, Fernando Valenzuela-Aedo, Rodrigo Lizama-Pérez, Máximo Escobar-Cabello

**Affiliations:** 1 Centro de Excelencia en Estudios Morfológicos y Quirúrgicos, Universidad de La Frontera, Av. Las Encinas 1000, Temuco, Chile; 2 Universidad de La Frontera, Programa de Doctorado en Ciencias Morfológicas, Av. Las Encinas, 1000 Temuco, Chile; 3 Hospital El Carmen de Maipú, camino a Rinconada 1201, Maipú, Chile; 4 Facultad de Medicina, Departamento de Ciencias de la Rehabilitación, Universidad de La Frontera, Claro Solar 115, Temuco, Chile; 5 Laboratory of Epidemiology and Morphological Sciences, Instituto de Biología, Pontificia Universidad Católica de Valparaíso, Av. Brasil 2950, Valparaíso, Chile; 6 Department of Physical Education and Sports, Faculty of Sport Sciences, University of Granada, 18011 Granada, Spain; 7 Laboratorio de Función Disfunción Ventilatoria, Departamento de Kinesiología, Universidad Católica del Maule, Av San Miguel 3605, Talca, Chile; 8 Universidad de La Frontera, Av. Francisco Salazar 01145, 4811230 Temuco, Chile

**Keywords:** COVID-19, Adult, Older, Pulmonary rehabilitation

## Abstract

**Background:**

It is internationally known that our population is aging. At the same time, some patients with COVID-19, due to their symptoms, required mechanical ventilation (MV) and subsequent pulmonary rehabilitation (PR). This study aimed to compare the effects of a multimodal PR program “ADULT” versus “OLDER” people with COVID-19 who were on MV.

**Methods:**

The intervention consisted of an 8-week hybrid PR program (2x week). Forced vital capacity (FVC) was measured at the beginning and end of PR, upper and lower limb strength was obtained through hand grip strength (HGS) and the sit-to-stand test (STST), respectively, and functional exercise capacity was measured with the 6-minute walking test (6MWT).

**Results:**

The main results were an increase in the FVC in the ADULT and OLDER groups (time effect, *P* = 0.000; η2 = 0.27), an increase in HGS in the ADULT and OLDER groups (time effect, *P* = 0.000; η2 = 0.52), in the same way, the number of repetitions on the STST increased in the ADULT and OLDER groups (time effect, *P* = 0.000; η2 = 0.55). Finally, the distance covered on the 6MWT increased in the ADULT and OLDER groups (time effect, *P* = 0.000; η2 = 0.65).

**Conclusions:**

The PR program is an effective strategy to improve FVC, muscle strength, and functional exercise capacity similarly in adults and older people with post severe COVID-19 who required MV.

## Background

All over the world, millions of people suffered from COVID-19-related severe acute respiratory syndrome [1, 2]. Due to their serious respiratory symptoms and, in some cases, acute respiratory difficulty, these patients required prolonged mechanical ventilation (MV) [3]. This prolonged use of MV can cause respiratory problems, cognitive problems, myopathies, neuropathies, physical deterioration, and cardiac disorders. In addition, COVID-19 is currently considered a multisystemic disease, where the major complications observed are in the respiratory and musculoskeletal systems [4–6]. In this sense, the American Thoracic Society-European Respiratory Society (ATS-ERS) suggests that after hospitalization, patients with COVID-19 who were connected to MV should enter a pulmonary rehabilitation (PR) program [7].

The intolerance to exercise is a “key symptom” that limits daily activities, resulting in an effort that goes beyond the typical to perform the basic activities of daily life [3, 8]. Thus, an assessment that gives information on the most compromised systems (respiratory and musculoskeletal) in the post-COVID-19 patient is fundamental. In this context, the evaluation of forced vital capacity (FVC), muscle strength tests, and the 6-minute walk test (6 MWT) are easily accessible and can provide a multisystemic view to enable better decision-making when addressing PR [7, 8].

It is internationally known that our population is aging [9]. A specific phenomenon that comes with aging is the loss of muscle mass, muscle strength, and physical performance, known as sarcopenia [10, 11], which is exacerbated by a pathological condition (i.e., COVID-19) or by physical inactivity as in the case of hospitalization with MV [10].

Considering this interaction between “aging” and COVID-19, Liu et al. (2020) analyzed the clinical characteristics of ADULT (< 60 years old) and OLDER (≥ 60 years old) patients hospitalized with pneumonia due to COVID-19. Their main results indicated that mortality in OLDER patients with COVID-19 is greater than in ADULT patients, and they also determined that the Pneumonia Severity Index was significantly higher in the OLDER than in the ADULT group (*p* < 0.001). They therefore concluded that OLDER people are more prone to developing more severe damage in the clinical picture [12]. Additionally, during the acute phase of the COVID-19 infection (∼ 2 weeks), the patient risks losing between 5 and 10% of their body weight, which is why the risk of acute sarcopenia is high. This could affect the hospital prognosis and lead to functional and physical deterioration post COVID-19 [13].

Therefore, in the present study, our objective was to compare the effects of a multimodal program of RP in “ADULT” versus “OLDER” people with COVID-19 who were connected to MV. We hypothesize that the FVC, muscle strength, and functional exercise capacity will increase after the PR program, but the impact will be smaller in the OLDER group than in the ADULT group.

## Methods

### Participants and experimental design

Ninety-eight healthy participants, 42 ADULT (< 60 years), 15/27 (female/male) 50 ± 6 years of age and body mass index (BMI) of 31.01 ± 4.23 kg/m^2^, and 56 OLDER (≥ 60 years), 22/34 (female/male) 66 ± 5 years of age and BMI of 29.69 ± 4.19 kg/m^2^. The study was conducted between September 2020 and March 2021 and was approved by the Scientific Ethics Committee of the Central Metropolitan Health Service, Chile (Resolution N° 378/2021). This study is part of a larger project with previous publications providing preliminary results or secondary analyses [14, 15]. All the participants were informed about the procedures of this study, agreed to participate, and signed the informed consent. The inclusion criteria were (a) COVID-19 diagnosis, (b) MV required, (c) medical hospital discharge, (d) check-up with a cardiologist and normal electrocardiogram, and (e) check-up with a bronchopulmonary specialist. The exclusion criteria were (a) patients that did not understand and/or could not follow orders and (b) BMI ≥ 40 kg/m^2^. All the volunteers undertook the PR program supervised by a physiotherapist, with a frequency of twice a week. Before and after the 8 weeks of training, spirometry was performed to obtain the FVC, a hand grip strength (HGS) test was used to determine the strength of the upper limbs, and the sit-to-stand test (STST) was used for the lower limbs and 6-MWT was used to determine functional exercise capacity.

### Measurements

**Forced Vital Capacity** For this, we used a Medgraphics spirometer (CPFS/D USB 2.02, MGC Diagnostics Corporation, Minnesota, USA). The following parameters were obtained: FVC, volume that has been exhaled at the end of the first second of forced expiration (FEV_1_), and the relation between the two (FEV_1_/FVC) [16].

#### Evaluation of muscle strength

The strength of the upper and lower limbs was evaluated using the HGS test and the STST. The HGS was measured with a Jamar hand® Plus + electronic dynamometer (Patterson Medical, Cedarburg, WI, USA) with the participant seated. Three attempts were made in each hand alternately with a 30-second rest. The highest value of the 6 attempts was reported [17]. The STST was done in a chair with no armrests or wheels, with the feet planted on the floor and the arms crossed over the chest. They had to rise fully from this position and return to the starting point as many times as possible in 30 s. The successful repetitions were reported [18].

#### Functional exercise capacity

The 6-MWT was performed in a straight 30-meter hallway. The participant walked the longest distance possible for the 6 min of the test. At the end of the test, the participant was notified, and the place where they stopped was marked to measure the distance covered in meters [19].

**Post-COVID-19 Functional Status (PCFS)** Functional status was measured with the PCFS, a scale with four questions to which the following graduation is assigned: grade 0: without functional limitations; grade 1: very slight functional limitations; grade 2: slight functional limitations; grade 3: moderate functional limitations; and grade 4: severe functional limitations. In the case where questions have the same functional limitation grade, the question with the greatest limitation grade is selected [20].

### Intervention

#### Pulmonary Rehabilitation Program

The sessions (2x/ week) were divided into 30 min of aerobic exercise, 20 min of strength exercise, and 10 min of flexibility, consisting of muscle stretching. The strength training of the inspiratory muscles was done at each patient’s home address (2x per day, 5x/ week). The PR program has been detailed in previous publications [14, 15].

### Statistical analysis

The data appear as mean ± standard deviation. The normal distribution of the data was determined with the Shapiro-Wilk test. The data between the groups were compared with the t-test for independent samples for the quantitative variables and with the Chi-squared test for the qualitative variables. The data before and after the intervention were analyzed with a repeated measures ANOVA with time as the within-subjects factor and the group (ADULT versus OLDER) as the between-subjects factor. In the case of a significant interaction, t-tests were performed where necessary. An α level of 0.05 was considered. In addition, the partial eta squared (η2) was used to estimate the effect sizes for the ANOVA and Cohen’s d for the comparison between two groups (d). The calculations were done with SPSS v. 24.0 (IBM Corp., Armonk, New York, USA).

## Results

### Participants

One hundred and thirty-three patients underwent a PR program. A subsample of 98 patients (according to inclusion/exclusion criteria), 42 participants in the ADULT group, and 56 participants in the OLDER group were considered in the present study, and all completed the evaluations included in the present report. Participants’ characteristics are shown in Table 1, observing a significant difference at baseline for age (*P* < 0.001), weight, and STST (*P* < 0.05).

**Table 1:** Comparison of demographic, spirometric and physical condition characteristics

	Adult (*n* = 42)	Older (*n* = 56)	p value
Gender (F/M)	15/27	22/34	-
Age (years)	50 ± 6	66 ± 5	0.0001^MW^
Weight (kg)	85.38 ± 14.56	79.09 ± 11.83	0.01^t^
Height (cm)	1.63 ± 0.07	1.62 ± 0.08	0.632^MW^
BMI (kg/m^2^)	31.01 ± 4.23	29.69 ± 4.19	0.092^MW^
**Hospitalization**			
MV connection (days)	18.70 ± 14.14	21.63 ± 14.09	0.194^MW^
PD duration (days)	3.19 ± 2.14	3.80 ± 2.97	0.174^MW^
Corticosteroids (n/%)	39/91	53/94	0.714
Sedation (n/%) 20/100	42/100	56/100	-
Neuromuscular blockade (n/%)	33/78	45/80	0.828
Home oxygen therapy (n/%)	3/7	6/10	0.544
**Measurements Initial**			
FVC (L)	3.12 ± 0.80	3.02 ± 0.80	0.507^t^
FVC (%Pred)	81.09 ± 15.51	91.12 ± 17.31	0.002^MW^
FEV_1_ (L/sec)	2.66 ± 0.68	2.46 ± 0.62	0.116^t^
FEV_1_ (%Pred)	86.54 ± 17.59	95.21 ± 16.32	0.020^MW^
FEV_1_/FVC (%)	85.17 ± 5.13	81.58 ± 5.62	0.0002^MW^
MIP (-cmH_2_O)	67.16 ± 24.35	62.85 ± 26.12	0.392^t^
6-MWT (m)	448.6 ± 100.1	422.4 ± 107.2	0.150^MW^
STST (rpm)	21.39 ± 5.77	18.98 ± 6.95	0.160^MW^
HGS right (kg)	21.15 ± 14.98	17.47 ± 9.52	0.229^MW^
HGS left (kg)	19.04 ± 13.71	15.88 ± 8.76	0.222^MW^
PCFS (Points)	3 (1–4)	3 (1–4)	0.940^MW^
**Morbid background**			
Obesity (n/%)	19/45.24	24/42.86	0.814
HTA (n/%)	18/42.86	45/80.36	0.0001
DM (n/%)	13/31	28/50	0.058

BMI: body mass index; MV: mechanical ventilator; PD: Prone Decubitus; FVC: forced vital capacity; FEV_1_: volume that has been exhaled at the end of the first second of forced expiration; MIP, maximal inspiratory pressure; 6MWT: 6-Minute Walk Test; STST: Sit-to-stand test; HGS: Hand grip strength; PCFS: Post COVID-19 Functional Status; t: Student’s t; MW: Mann Whitney

### Forced vital capacity

**Fig. 1:**
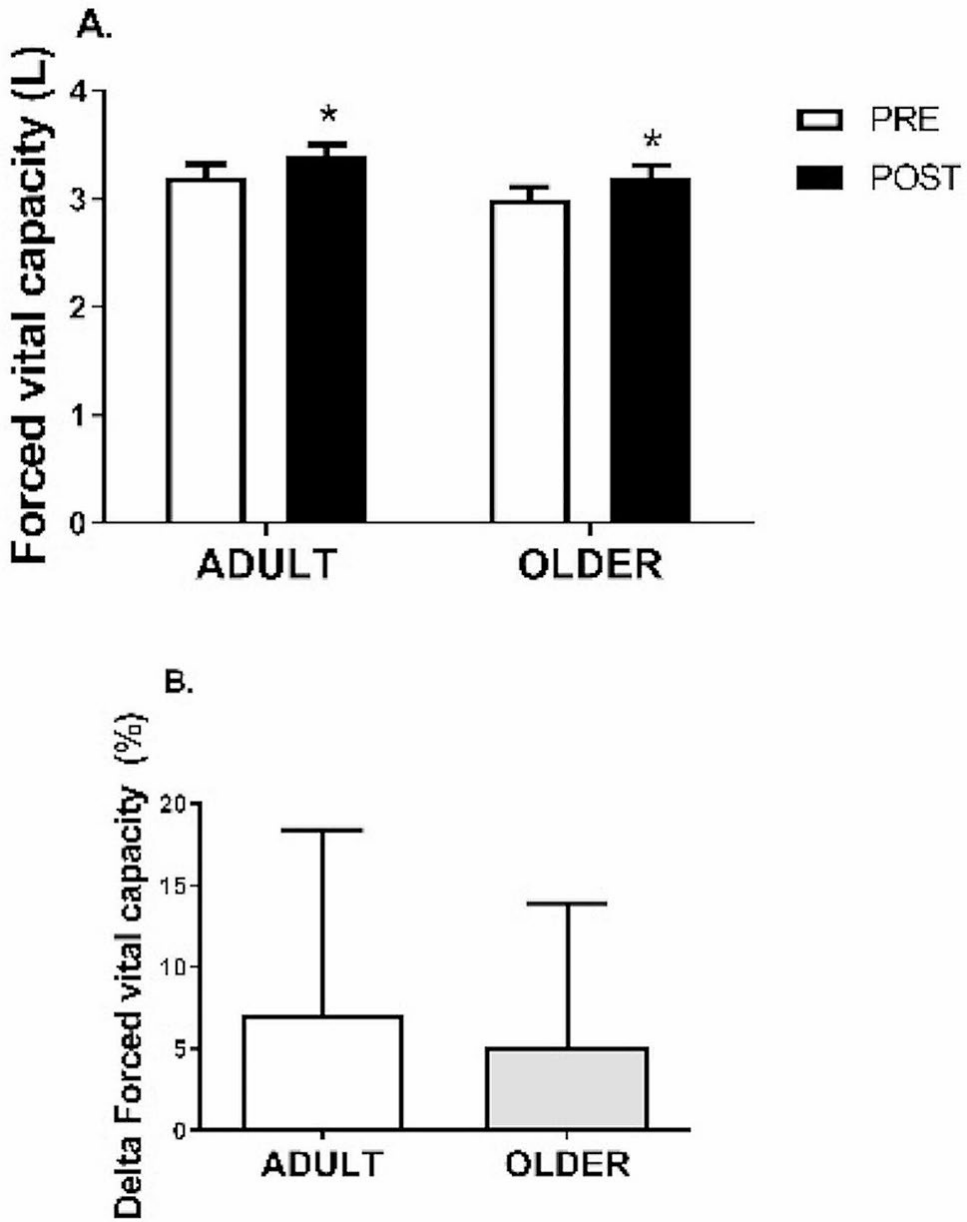
Forced vital capacity (**A**) Variation between PRE and POST of ADULT and OLDER groups (**B**) Comparison of the change percentage between ADULT and OLDER. Participants in ADULT group, *n* = 42, and OLDER group, *n* = 56. The data were analyzed using a repeated measures ANOVA (**A**) and an independent samples t-test (**B**) * *p* < 0.05 (time effect)

After the PR program, FVC (Fig. 1) increased from 3.2 ± 0.8 to 3.4 ± 0.7 L (7.1 ± 11.3%) in the ADULT group and from 3.0 ± 0.8 to 3.2 ± 0.8 L (5.1 ± 8.8%) in the OLDER group (time effect, *P* = 0.000; η2 = 0.27) (Table 2). No differences in response to the PR program were observed between groups in FVC (time*group interaction effect, *P* = 0.441; η2 = 0.006) (Table 2). Accordingly, the delta increase in FVC did not differ between ADULT and OLDER (*P* > 0.05).

**Table 2:** Comparison between ADULT and OLDER, of spirometric and physical condition variables and changes after pulmonary rehabilitation

	ADULT (*n* = 42)		OLDER (*n* = 56)				
	**PRE**	**POST**	**PRE**	**POST**	**Time**	Time*Group	**Group**
FVC (L)	3.12 ± 0.80	3.31 ± 0.73	3.02 ± 0.80	3.15 ± 0.82	0.000	0.441	0.166
FVC (%Pred)	81.09 ± 15.51	86.09 ± 13.29	91.12 ± 17.31	94.47 ± 15.51	0.000	0.373	0.015
FEV_1_ (L/sec)	2.66 ± 0.68	2.78 ± 0.62	2.46 ± 0.62	2.54 ± 0.64	0.000	0.417	0.017
FEV_1_ (%Pred)	86.54 ± 17.59	90.89 ± 15.26	95.21 ± 16.32	98.96 ± 14.35	0.000	0.715	0.036
FEV_1_/CVF (%)	85.17 ± 5.13	84.09 ± 5.78	81.58 ± 5.62	81.56 ± 6.35	0.078	0.089	0.002
MIP (-cmH_2_O)	67.16 ± 24.35	81.53 ± 26.63	62.85 ± 26.12	74.36 ± 26.66	0.000	0.394	0.151
6MWT (m)	448.6 ± 100.1	538.2 ± 75.2	422.4 ± 107.2	492.2 ± 99.26	0.000	0.070	0.019
STST (rpm)	21.39 ± 5.77	27.11 ± 5.61	18.98 ± 6.95	23.12 ± 6.00	0.000	0.111	0.022
HGS right	21.15 ± 14.98	27.54 ± 17.44	17.47 ± 9.52	22.11 ± 11.28	0.000	0.040	0.031
HGS left	19.04 ± 13.71	25.65 ± 17.17	15.88 ± 8.76	19.93 ± 11.32	0.000	0.009	0.028

FVC: forced vital capacity; FEV_1_: volume that has been exhaled at the end of the first second of forced expiration; MIP, maximal inspiratory pressure; 6-MWT: 6-Minute Walk Test; STST: Sit-to-stand test; HGS: Hand grip strength

### Strength

**Fig. 2:**
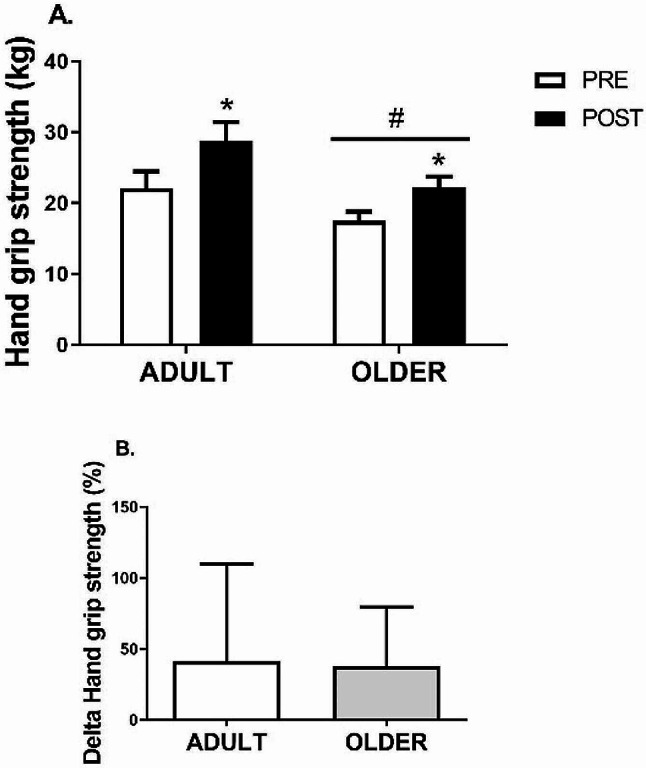
Hand grip strength. (**A**) Variation between PRE and POST of ADULT and OLDER groups (**B**) Comparison of the change percentage between ADULT and OLDER. Participants in ADULT group, *n* = 42, and OLDER group, *n* = 56. The data were analyzed using a repeated measures ANOVA (**A**) and an independent samples t-test (**B**) * *p* < 0.05 (time effect)

**Fig. 3:**
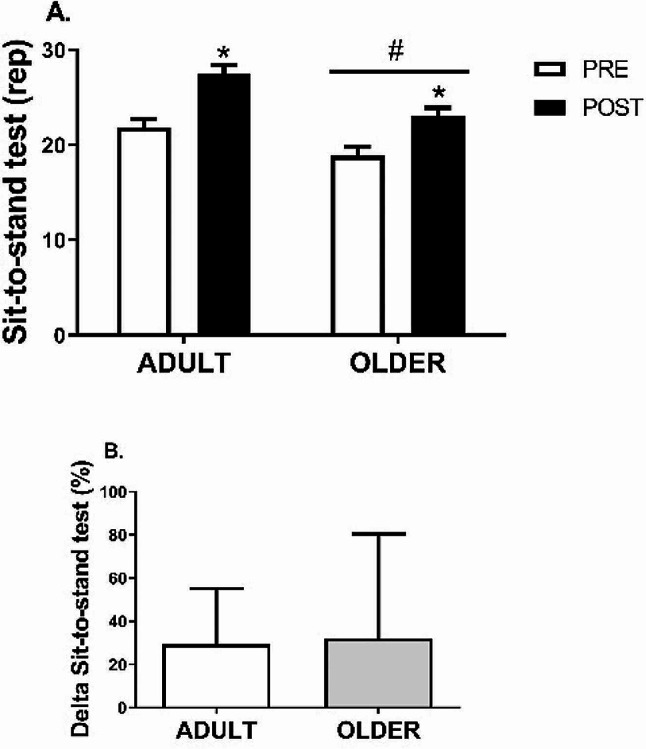
Sit-to-stand test. (**A**) Variation between PRE and POST of ADULT and OLDER groups (**B**) Comparison of the change percentage between ADULT and OLDER. Participants in ADULT group, *n* = 42, and OLDER group, *n* = 56. The data were analyzed using a repeated measures ANOVA (**A**) and an independent samples t-test (**B**) * *p* < 0.05 (time effect)

After the PR program, HGS (Fig. 2) increased from 22.1 ± 15.3 to 28.7 ± 17.7 kg (41.6 ± 68.5%) in the ADULT group and from 17.5 ± 9.6 to 22.2 ± 11.4 kg (37.7 ± 41.8%) in the OLDER group (time effect, *P* = 0.000; η2 = 0.52) (Table 2). Similarly, 8 weeks of the PR program resulted in an increase in leg strength (Fig. 3) from 21.9 ± 5.6 to 27.5 ± 5.7 rep (29.3 ± 25.8%) in the ADULT group and from 18.9 ± 7.0 to 23.1 ± 6.0 rep (32.0 ± 48.5%) in the OLDER group (time effect, *P* = 0.000; η2 = 0.55) (Table 2). Differences between the ADULT and OLDER groups were observed in grip and leg strength (group effect, *P* = 0.002; η2 = 0.09 and *P* = 0.041; η2 = 0.04, respectively) (Table 2). No differences in response to the PR program were observed between groups in any strength variables (time*group interaction effect, all *P* > 0.092; all η2 ≤ 0.29) (Table 2). Accordingly, the delta increase in grip and leg strength did not differ between the ADULT and OLDER groups (*P* > 0.05).

### Functional exercise capacity

**Fig. 4:**
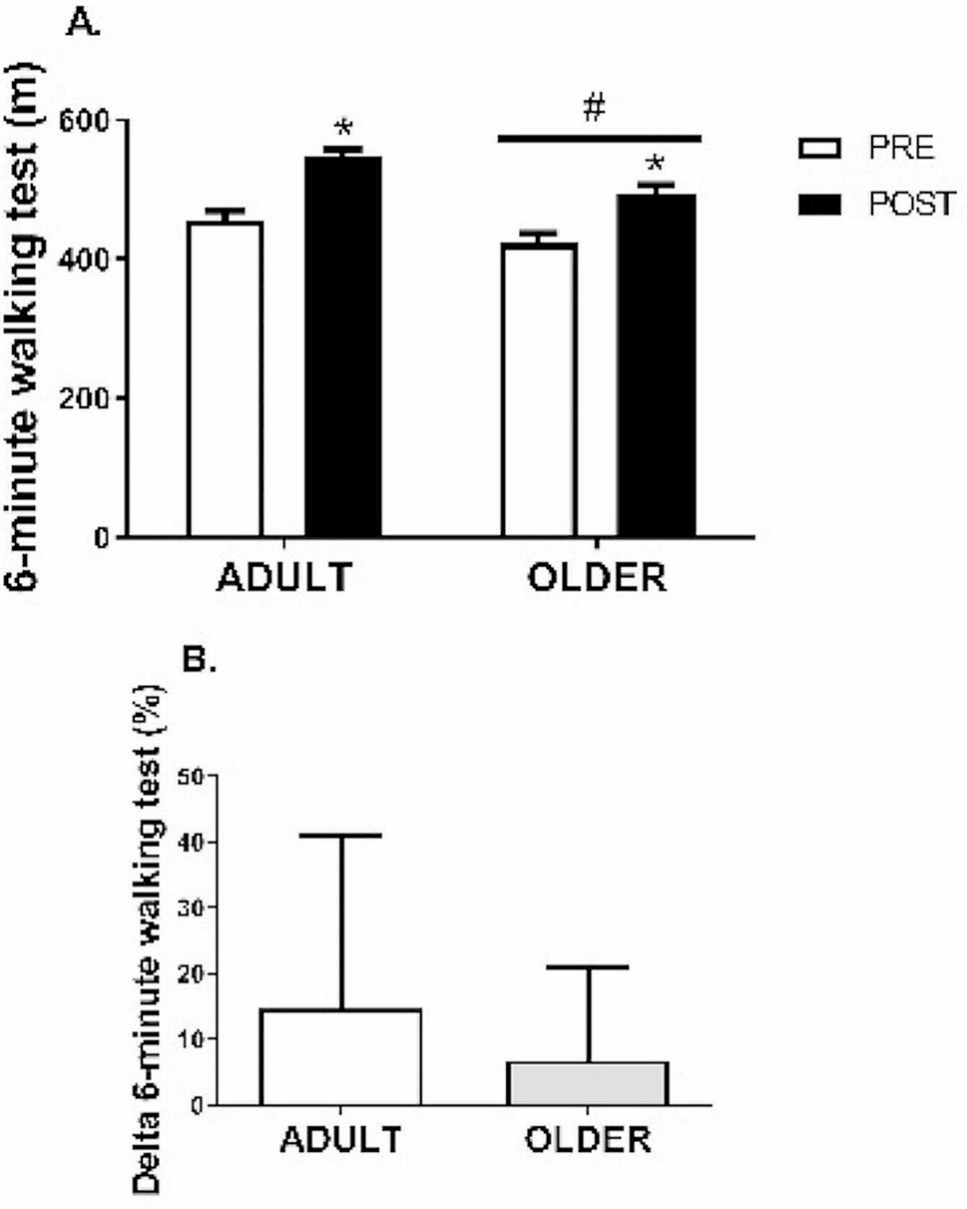
6-Minute Walking Test. (**A**) Variation between PRE and POST of ADULT and OLDER groups (**B**) Comparison of the change percentage between ADULT and OLDER. Participants in ADULT group, *n* = 42, and OLDER group, *n* = 56. The data were analyzed using a repeated measures ANOVA (**A**) and an independent samples t-test (**B**) * *p* < 0.05 (time effect)

The PR program increased functional exercise capacity (Fig. 4) from 455.0 ± 93.1 to 548.2 ± 62.2 m in the ADULT and from 422.9 ± 108.1 to 493.7 ± 99.5 m in the OLDER group (time effect, *P* = 0.000; η2 = 0.65) (Table 2). Differences between groups were observed between ADULT and OLDER groups in functional exercise capacity (group effect, *P* = 0.019; η2 = 0.06) (Table 2). No differences in response to the PR program were observed between groups in functional exercise capacity (time*group interaction effect, *P* = 0.070; η2 = 0.03) (Table 2). Accordingly, the relative increase in functional exercise capacity did not differ between the ADULT (25.1 ± 28.0%) and OLDER groups (21.5 ± 35.2%; *P* > 0.05).

## Discussion

This study aimed to compare the effects of a multimodal PR program in “ADULT” versus “OLDER” people with COVID-19 who had been connected to MV. The main results indicate that the proposed PR program significantly improves FVC, muscle strength, and functional exercise capacity. Moreover, no significant differences were observed when comparing the improvement percentage between the ADULT and OLDER groups.

There is ample existing evidence to indicate the impact of a PR program on spirometric variables, muscle strength, and exercise capacity. Specifically, Hayden et al. (2021) analyzed the effects of a PR program on dyspnea and other relevant clinical variables in patients post-COVID-19 of varying severity. Their results led them to conclude that a PR program could be safe, feasible, and effective in patients post COVID-19 after the acute stage, thereby improving their pulmonary function, strength, and quality of life [21]. Thus, PR has proven to be an intervention model with multisystemic impact, which generates significant improvements in the cardiorespiratory and musculoskeletal systems, regardless of the severity of the clinical picture [14, 15].

Previously we indicated that changes in the musculoskeletal and cardiorespiratory systems accompany aging. These changes have proven to be related to each other. Miyatake et al. (2022) studied the association between muscle strength of the limbs and the percent predicted FVC and how this would affect the mortality of Japanese adults. Their results indicated that men with a lower percent predicted FVC have a greater mortality risk (hazard ratio [HR] = 2.03); when adding lower limb strength, the mortality risk was modified (HR = 1.78). On the other hand, in women only the reduction of the percent predicted FVC was a mortality risk factor (HR = 1.67). In conclusion, a low percent predicted FVC was associated with high mortality and unfavorable effects of lower limb strength in men [22]. The results of the present study indicated that in older adults, a significant reduction in FVC, distance covered on the 6MWT, and limb strength was observed. However, after the PR program, these variables increased significantly. This is important, considering that a low FVC value, added to poor lower limb muscle strength, is linked to a high mortality risk.

PR in the ADULT and OLDER groups has proven to have good effects. Specifically, Liu et al. (2020) investigated the effects of a 6-week PR program on pulmonary function, exercise capacity, and quality of life in OLDER patients with COVID-19. Their results indicated a significant increase in pulmonary function, exercise capacity, and quality of life, except for the domains of anxiety and depression, compared to the control group [23]. In the same way, Araújo et al. (2023) studied the effects of cardiopulmonary rehabilitation consisting of aerobic and strength exercises of moderate intensity in post COVID-19 adult patients. Their results indicated that the PR improved pulmonary function, respiratory muscle strength, tolerance to maximal and submaximal exercise, and quality of life of post COVID-19 patients [24]. In this context, the results obtained in this study, in addition to agreeing with the existing evidence, contribute to the comparison of the change percentage between the ADULT and OLDER groups in severe post COVID 19 patients who required MV.

Another important result was the absence of significant differences in the improvement percentage between the ADULT and OLDER groups in the variables FVC, HGS, STST and distance covered. This agrees with the report by Marzuca-Nasrr et al. (2022), who compared the effects of a cardiac rehabilitation hybrid program in “ADULT” vs. “OLDER” people with coronary arteriopathy. In addition, they hypothesized that the cardiac rehabilitation hybrid program would have less impact on the older group than on adult individuals. Their results indicate an improvement following the rehabilitation program in both groups, with no difference in the percentage of variation when the ADULT and OLDER groups were compared [25]. Although the results of the present study are consistent with previous reports, the comparison is on different clinical pictures. This fact reinforces that exercise is favorable to maintaining and increasing functionality regardless of the normal process of reducing muscle mass, strength, and physical performance inherent to aging and some particular pathologies/conditions.

This study has limitations that are important to mention. As a substudy, it considers participants included in a project of broader scope with another objective. Moreover, the “balance” between variables could create a bias that is difficult to manage (i.e., gender per group, etc.).

## Conclusions

A PR program is an effective strategy to improve FVC, muscle strength, and functional exercise capacity similarly in ADULTS and OLDER people with post severe COVID-19 who required MV.

## Data Availability

The datasets used and/or analysed during the current study available from the corresponding author on reasonable request.

## References

[1] Tiryaki Ş, Dabeşlim H, Aksu Y. Chest computed tomographic findings of patients with COVID-19-related pneumonia. Acta Radiol Open. 2021;10(2):2058460121989309. 33614161 10.1177/2058460121989309 PMC7874355

[2] Aksu Y, Uslu AU, Tarhan G, Karagülle M, Tiryaki Ş. The relationship among Splenomegaly, Lung involvement patterns, and severity score in COVID-19 Pneumonia. Curr Med Imaging. 2022;18(12):1311–7. 35579138 10.2174/1573405618666220509212035

[3] Del Valle MF, Valenzuela J, Godoy L, Del Sol M, Lizana PA, Escobar-Cabello M, Muñoz-Cofre R. Letter Chile Respirol. 2022;27(2):173–74. 10.1111/resp.14196 34913226

[4] Horton R, Offline. COVID-19 is not a pandemic. Lancet. 2020;396(10228):935. 32979964 10.1016/S0140-6736(20)32000-6 PMC7515561

[5] Wang TJ, Chau B, Lui M, Lam GT, Lin N, Humbert S. Physical Medicine and Rehabilitation and Pulmonary Rehabilitation for COVID-19. Am J Phys Med Rehabil. 2020;99(9):769–74. 32541352 10.1097/PHM.0000000000001505 PMC7315835

[6] Alvarez R, Del Valle MF, Cordero P, del Sol M, Lizana PA, Gutierrez J, Valenzuela J, Munoz-Cofré R. Shoulder Pain in COVID-19 survivors following mechanical ventilation. Int J Environ Res Public Health. 2021;18(19):10434. 34639733 10.3390/ijerph181910434 PMC8507893

[7] Spruit MA, Holland AE, Singh SJ, Tonia T, Wilson KC, Troosters T. COVID-19: Interim Guidance on Rehabilitation in the Hospital and Post-hospital Phase from a European Respiratory Society and American Thoracic Society-Coordinated International Task Force. Eur Respir J. 2020;56(6):2002197. 32817258 10.1183/13993003.02197-2020 PMC7427118

[8] Daher A, Balfanz P, Cornelissen C, Müller A, Bergs I, Marx N, Müller-Wieland D, Hartmann B, Dreher M, Müller T. Follow up of patients with severe coronavirus disease 2019 (COVID-19): Pulmonary and extrapulmonary disease sequelae. Respir Med. 2020;174:106197. 33120193 10.1016/j.rmed.2020.106197 PMC7573668

[9] Khan MA, Hashim MJ, Mustafa H, Baniyas MY, Al Suwaidi S, Alkatheeri R, Alblooshi FMK, Almatrooshi MEAH, Alzaabi MEH, Al Darmaki RS, et al. Global epidemiology of ischemic heart disease: results from the global burden of disease study. Cureus. 2020;12(7):e9349. 32742886 10.7759/cureus.9349 PMC7384703

[10] Aguiar GB, Dourado KF, Andrade MIS, Domingos Júnior IR, Barros-Neto JA, Vasconcelos SML, Petribú MMV, Santos CMD, Moura MWS, Aguiar CB, et al. Frequency and factors associated with Sarcopenia prediction in adult and elderly patients hospitalized for COVID-19. Exp Gerontol. 2022;168:111945. 36064158 10.1016/j.exger.2022.111945 PMC9443615

[11] Rudnicka E, Napierala P, Podfigurna A, Meczekalski B, Smolarczyk R, Grymowicz M. The world health organization (who) approach to healthy ageing. Maturitas. 2020;139:6–11. 32747042 10.1016/j.maturitas.2020.05.018 PMC7250103

[12] Liu K, Chen Y, Lin R, Han K. Clinical features of COVID-19 in elderly patients: a comparison with young and middle-aged patients. J Infect. 2020;80(6):e14–8. 32171866 10.1016/j.jinf.2020.03.005 PMC7102640

[13] Piotrowicz K, Gąsowski J, Michel JP, Veronese N. Post-COVID-19 acute sarcopenia: physiopathology and management. Aging Clin Exp Res. 2021;33(10):2887–98. 34328636 10.1007/s40520-021-01942-8 PMC8323089

[14] Del Valle MF, Valenzuela J, Marzuca-Nassr GN, Cabrera-Inostroza C, del Sol M, Lizana PA, Escobar-Cabello M, Muñoz-Cofré R. Eight weeks of supervised Pulmonary Rehabilitation are effective in improving resting heart rate and heart rate recovery in severe COVID-19 patient survivors of mechanical ventilation. Med (Kaunas). 2022;58(4):514. 10.3390/medicina58040514 PMC9028941 35454353

[15] Del Valle MF, Valenzuela J, Marzuca-Nassr GN, Godoy L, Del Sol M, Lizana PA, Escobar-Cabello M, Muñoz-Cofré R. Use of the speed achieved on the 6MWT for programming aerobic training in patients recovering from severe COVID-19: an observational study. Ann Med. 2023;55(1):889–97. 36881045 10.1080/07853890.2023.2179658 PMC10795638

[16] Graham BL, Steenbruggen I, Miller MR, Barjaktarevic IZ, Cooper BG, Hall GL, Hallstrand TS, Kaminsky DA, McCarthy K, McCormack MC, et al. Standardization of Spirometry 2019 Update. An official American Thoracic Society and European Respiratory Society Technical Statement. Am J Respir Crit Care Med. 2019;200(8):e70–88. 31613151 10.1164/rccm.201908-1590ST PMC6794117

[17] Roberts HC, Denison HJ, Martin HJ, Patel HP, Syddall H, Cooper C, Sayer AA. A review of the measurement of grip strength in clinical and epidemiological studies: towards a standardised approach. Age Ageing. 2011;40(4):423–29. 21624928 10.1093/ageing/afr051

[18] Rikli RE, Jones CJ. Development and validation of criterion-referenced clinically relevant fitness standards for maintaining physical independence in later years. Gerontologist. 2013;53(2):255–67. 22613940 10.1093/geront/gns071

[19] Muñoz-Cofré R, Medina-González P, Escobar-Cabello M. Análisis Del comportamiento temporal de variables fisiológicas y de esfuerzo en sujetos instruidos en la Prueba De Caminata en 6 minutos: Complemento A La Norma ATS. Fisioterapia. 2016;38(1):20–7.

[20] Klok FA, Boon GJAM, Barco S, Endres M, Geelhoed JJM, Knauss S, Rezek SA, Spruit MA, Vehreschild J, Siegerink B. The Post-COVID-19 functional status scale: a tool to measure functional status over time after COVID-19. Eur Respir J. 2020;56(1):2001494. 32398306 10.1183/13993003.01494-2020 PMC7236834

[21] Hayden MC, Limbach M, Schuler M, Merkl S, Schwarzl G, Jakab K, Nowak D, Schultz K. Effectiveness of a three-week Inpatient Pulmonary Rehabilitation Program for patients after COVID-19: a prospective observational study. Int J Environ Res Public Health. 2021;18(17):9001. 34501596 10.3390/ijerph18179001 PMC8430843

[22] Miyatake M, Okazaki T, Suzukamo Y, Matsuyama S, Tsuji I, Izumi S-I. High mortality in an older Japanese Population with low forced vital capacity and gender-dependent potential impact of muscle strength: longitudinal cohort study. J Clin Med. 2022;11(18):5264. 36142910 10.3390/jcm11185264 PMC9505108

[23] Liu K, Zhang W, Yang Y, Zhang J, Li Y, Chen Y. Respiratory rehabilitation in elderly patients with COVID-19: a randomized controlled study. Complement Ther Clin Pract. 2020;39:101166. 32379637 10.1016/j.ctcp.2020.101166 PMC7118596

[24] Araújo BTS, Barros AEVR, Nunes DTX, Remígio de Aguiar MI, Mastroianni VW, de Souza JAF, Fernandes J, Campos SL, Brandão DC, Dornelas, de Andrade A. Effects of continuous aerobic training associated with resistance training on maximal and submaximal exercise tolerance, fatigue, and quality of life of patients post-COVID-19. Physiother. Res. Int. 2023;28(1):e1972. 10.1002/pri.1972 PMC9539049 36088642

[25] Marzuca-Nassr GN, Seron P, Román C, Gálvez M, Navarro R, Latin G, Marileo T, Molina JP, Sepúlveda P, Oliveros MJ. A hybrid exercise-based cardiac rehabilitation program is an effective strategy to improve muscle strength and functional exercise capacity in adults and older people with coronary artery disease. Front Physiol. 2022;13:948273. 35991183 10.3389/fphys.2022.948273 PMC9389047

