# Correction: A pulmonary rehabilitation program is an effective strategy to improve forced vital capacity, muscle strength, and functional exercise capacity similarly in adults and older people with post-severe COVID-19 who required mechanical ventilation

**DOI:** 10.1186/s12877-024-05051-9

**Published:** 2024-10-22

**Authors:** Rodrigo Muñoz-Cofré, María Fernanda del Valle, Gabriel Nasri Marzuca-Nassr, Jorge Valenzuela, Mariano del Sol, Constanza Díaz Canales, Pablo A. Lizana, Fernando Valenzuela-Aedo, Rodrigo Lizama-Pérez, Máximo Escobar-Cabello

**Affiliations:** 1 Centro de Excelencia en Estudios Morfológicos y Quirúrgicos, Universidad de La Frontera, Av. Las Encinas, Temuco, 1000 Chile; 2 Universidad de La Frontera, Programa de Doctorado en Ciencias Morfológicas, Av. Las Encinas, Temuco, 1000 Chile; 3 Hospital El Carmen de Maipú, camino a Rinconada 1201, Maipú, Chile; 4 Facultad de Medicina, Departamento de Ciencias de la Rehabilitación, Universidad de La Frontera, Claro Solar 115, Temuco, Chile; 5 Laboratory of Epidemiology and Morphological Sciences, Instituto de Biología, Pontificia Universidad Católica de Valparaíso, Av. Brasil 2950, Valparaíso, Chile; 6 Department of Physical Education and Sports, Faculty of Sport Sciences, University of Granada, 18011 Granada, Spain; 7 Laboratorio de Función Disfunción Ventilatoria, Departamento de Kinesiología, Universidad Católica del Maule, Av San Miguel 3605, Talca, Chile; 8 Universidad de La Frontera, Av. Francisco Salazar 01145, Temuco, 4811230 Chile

**BMC Geriatrics (2024) 24:313**

10.1186/s12877-024-04910-9

After publication of this article, the authors reported that the details for affiliation 5 were incorrectly given as ‘Laboratory of Epidemiology and Morphological Sciences, Instituto de Biología, Universidad Católica de Valparaíso, Av. Brasil 2950, Valparaíso, Pontificia, Chile’ but should have been ‘Laboratory of Epidemiology and Morphological Sciences, Instituto de Biología, Pontificia Universidad Católica de Valparaíso, Av. Brasil 2950, Valparaíso, Chile’.

The original article has been corrected.

